# CLINICALLY MEANINGFUL CHANGE FOR PHYSICAL PERFORMANCE: PERSPECTIVES OF THE ICFSR TASK FORCE

**DOI:** 10.14283/jfa.2019.33

**Published:** 2020

**Authors:** J. GURALNIK, K. BANDEEN-ROCHE, S.A.R. BHASIN, S. EREMENCO, F. LANDI, J. MUSCEDERE, S. PERERA, J.-Y. REGINSTER, L. WOODHOUSE, B. VELLAS, THE ICFSR TASK FORCE

**Affiliations:** 1 University of Maryland School of Medicine, Baltimore, MD, USA; 2 Johns Hopkins Bloomberg School of Public Health, Baltimore, MD, USA; 3 Brigham and Women’s Hospital, Harvard Medical School, Boston, MA, USA; 4 Critical Path Institute, Tucson, AZ, USA; 5 Fondazione Policlinico A. Gemelli, Catholic University, Rome, Italy; 6 Queen’s University, Kingston, Ontario, Canada; 7 University of Pittsburgh, Pittsburgh, PA, USA; 8 University of Liege, Liege, Belgium; 9 University of Alberta, Edmonton, Alberta, Canada;; 10 Gerontopole, INSERM U1027, Alzheimer’s Disease Research and Clinical Center, Toulouse University Hospital, Toulouse, France

**Keywords:** Sarcopenia, frailty, aging, disability, physical performance, clinically meaningful change, outcome measures

## Abstract

For clinical studies of sarcopenia and frailty, clinically meaningful outcome measures are needed to monitor disease progression, evaluate efficacy of interventions, and plan clinical trials. Physical performance measures including measures of gait speed and other aspects of mobility and strength have been used in many studies, although a definition of clinically meaningful change in performance has remained unclear. The International Conference on Frailty and Sarcopenia Research Task Force (ICFSR-TF), a group of academic and industry scientists investigating frailty and sarcopenia, met in Miami Beach, Florida, USA in February 2019 to explore approaches for establishing clinical meaningfulness in a manner aligned with regulatory authorities. They concluded that clinical meaningful change is contextually dependent, and that both anchor-based and distribution-based methods of quantifying physical function are informative and should be evaluated relative to patient-reported outcomes. In addition, they identified additional research needed to enable setting criteria for clinical meaningful change in trials.

## Introduction

Clinical research studies in older populations have shifted over the last two decades from assessing biological indicators and disease status to measuring physical function as a primary endpoint. This shift reflects the World Health Organization’s (WHO’s) framework for health and disability, the International Classification of Functioning, Disability and Health (ICF) (1), which provides a multidimensional framework for conceptualizing and understanding functioning and disability by integrating medical and biopsychosocial models. Using a patient-focused approach, the ICF promotes the use of language that frames disablement not in terms of disease but in how people live with their conditions across three domains: body function and structure, activity, and participation, i.e., movement in three-dimensional space, interacting with other people, and socialization (2).

With new interventions for sarcopenia on the horizon, the concept of clinical meaningfulness has emerged as an important concern for researchers, clinicians, and regulators. Thus, the International Conference on Frailty and Sarcopenia Research Task Force (ICFSR-TF), a group of academic and industry scientists investigating frailty and age-related muscle loss (sarcopenia), convened a group of experts on February 19, 2019 to explore approaches for establishing clinical meaningfulness and related regulatory considerations.

Clinical meaningfulness, as defined by the U.S. Food and Drug Administration (FDA), requires that an outcome assessment measure something that is clinically important and that substantively affects how the patient feels, functions, or survives. Thus, clinically meaningful outcome measures for sarcopenia and frailty typically assess physical function, quality of life, and survival. Muscle strength and muscle mass may also be considered as outcome measures but only if they correlate with improved function or predict other relevant health outcomes such as reduced mortality, morbidity, institutionalization, and disability (3–7).

Clinically meaningful measures may be used to monitor adults in clinical settings and in observational studies, to evaluate efficacy in clinical trials, and to compute sample size and power when planning a clinical trial (8). However, meaningful change in an observational study may differ from meaningful change in an intervention trial where change can occur much more rapidly in the positive direction and must have both clinical and public health relevance. Since rapid changes may be perceived as being much greater in magnitude than those that occur more gradually, objective measurement is important.

## Defining a clinically meaningful change in physical performance

Meaningful change can be defined as a change that has clinical or practical importance, has an impact on an individual’s self-perceived health status or quality of life, or as a fraction of the standard deviation representing a certain level of movement across the distribution of measurements in the population. Clinically meaningful change depends on the outcome on which it is based. Physical performance measures regularly used in clinical trials include various measures of gait and balance parameters and/or the Short Physical Performance Battery (SPPB), a composite measure of walking speed, standing balance, and sit-to-stand performance (9). Gait performance measures include the 4-meter gait speed test (4MGS), the 6-minute walk distance test (6MWD), the 10-meter walk test (10MWT), the timed 400-meter walk (400MW), and the 3-meter timed “Up & Go” test (TUG) (10), (11). Other possible measures such as gait variability, unipodal balance, and stair negotiation performance may also be used to assess mobility impairments (12, 13). Most evidence has been gathered for the 4MGS, which can be performed in a reasonably small space. For example, in a prospective cohort study of older adults, Perera and colleagues showed that a decline in gait speed of 0.1 m/s on the 4MGS or 1 point on the SPPB over a one-year period was associated with an increased risk of subsequent mortality (14).

Clinically meaningful changes in outcomes may be expressed as changes that exceed minimally clinically important differences (MCID), clinically meaningful differences (CMD), or minimally important changes (MIC) (15). To determine the MCID and Minimally Clinically Important Improvement (MCII), either distribution-based or anchor-based measures may be used. Distribution-based methods use statistical and psychometric properties of a measure to estimate effect size and standard error of measurement (SEM=σ(1-r)^1/2^, where σ=standard deviation and r=reliability (16)) as functions of variability and reliability, while anchor-based methods use a change in the patient’s or provider’s perception to identify the corresponding magnitude of change in a selected measure (8).

Preliminary work by Perera and colleagues estimated what constitutes a meaningful change for three performance measures: gait speed, SPPB, and 6MWD using data from varying populations enrolled in both observational and clinical studies: 1) a basic training data set of a 3-month clinical trial of strength training intervention in people with mild-to-moderate limitations; 2) 1-year data of participants in the Predicting Elderly Performance (PEP) study dataset; and 3) 3-month data from the Stroke Rehabilitation (REHAB) randomized clinical trial of a therapeutic exercise program (8). Using both distribution- and anchor-based approaches, they concluded that small but meaningful changes are near to 0.05 m/s for gait speed, 0.5 points for SPPB, and 20m for 6MWD; and that substantial changes were near to 0.10 m/s for gait speed, 1.0 point for SPPB, and 50m for 6MWD.

They also found that meaningful changes are not affected by gender, race, or baseline performance in the Health ABC study. While men tended to have greater magnitudes for meaningful change in 400MWT and there were health and disease differences (e.g. substantial change estimate for SPPB for those with greater body mass index (BMI) when the anchor of walking ¼ mile was used, but not using other anchors), they did not show a consistent pattern and were limited by dropout bias in 400MWT (17).

In the Lifestyle Interventions and Independence for Elders Pilot (LIFE-P) study of exercise as an intervention, investigators examined the relationship between self-reported and performance measures and estimated the magnitude of meaningful change in 400MWT, 4MGS, and SPPB (18). They used both distribution-based and anchor-based methods, two magnitudes of change, and multiple indicators of self-perceived mobility. Relationships between self-reported and performance measures were consistent between treatment arms. Minimally significant changes were 20–30 seconds in the 400MWT, 0.03–0.05 m/s in the 4MGS, and 0.3–0.8 points in the SPPB. Substantial changes were 50–60 seconds in the 400MWT, 0.08 m/s in the 4MGS, and 0.4–1.5 points in the SPPB.

## A validation approach to define meaningful change

A crucial first step in defining meaningful change is to clarify what is meant by the concept of meaningful change. A clinically important change in physical functioning should be large enough that a person perceives the change or that participation (e.g., daily roles) is affected. In clinical trials, a clinically important change indicates a treatment effect large enough to support market authorization of a drug. The analytical approach chosen should be driven by how meaningful change is defined for a particular study depending on its main purpose.

Defining meaningful change may be challenging for several reasons. First, meaningful change varies according to context, including baseline level of function as well as demographic and disease considerations. Second, when no gold standard exists with which to make a comparison, the measures by which meaningful changes are judged may not reflect the true state.

One method for assessing the ability of a measure to discriminate individuals by their anchor status is to determine meaningful adverse change (MAC) that achieves both good sensitivity and specificity (19). The Women’s Health and Aging Study (WHAS), an observational study on the characteristics and progression of disability in older, functionally limited women (20) provides an example of a validation framework for evaluating change over the course of one year using usual pace walking speed as the performance measure and self-reported walking difficulties as the anchor. Participants were dichotomized into those who worsened in any one of seven categories of walking difficulty and those who did not worsen in any category, and walking speed change was compared for those two groups. The difference in mean change between those two groups was estimated at −0.091 meters/sec (95% confidence interval [CI] of −0.128 to −0.054), with a mean change among those not worsening of 0.011 (95% CI of −0.014 to 0.035). A decline of 0.10 m/sec (substantial change), however, had a sensitivity of .41 and specificity of 0.73 for self-perceived worsening, and receiver operating characteristic (ROC) analysis of the ability to discriminate clinical change yielded an area under the curve (AUC) of only 0.59, suggesting that other considerations would be needed to adjudicate whether this is good enough for clinical practice in the community-dwelling context of the WHAS. Reanalyzing the data by evaluating empirical cumulative probability distributions of walking speed stratified by decline in 3 categories of walking difficulty all the way to improving in 3 categories of walking difficulty yielded overlapping curves (except when contrasting perception changes transitioning across multiple categories), indicating that either the anchor is inappropriate or a more sensitive performance measure is needed. In such a context, building performance indices combining multiple measures simultaneously may prove useful for enhancing precision.

## Combining performance and patient reported outcome measures

Patient reported outcome measures (PROMs) have been advocated by regulatory agencies because they provide information about what is meaningful to patients. For example, fear of falling is one possible patient-reported measure that might correlate well with balance, strength, and other mobility-related functions. Many studies combine PROMs with performance measures since they provide complementary information (21). In a prospective cohort study, Perera and colleagues showed that performance change and self-reported change were both independently associated with 5-year survival (14).

Studies comparing self-reported versus activity-based performance measures of function such as self-paced walk, TUG, and stair tests have shown moderate correlations (22–25), suggesting that the measures provide complementary information. Moreover, these studies show that the selection of measures is condition specific. For example, in these studies the TUG was shown to be the most sensitive measure to change in patients who have undergone total hip replacement, while in patients undergoing knee arthroplasty the stair measure was more responsive to change.

## Case study: Determining meaningful change in physical function in testosterone trials in older men (TOM)

The Testosterone in Older Men with Mobility Limitations (TOM) trial was designed to determine the effect of testosterone administration on physical function and lower extremity strength in older men with mobility limitations and low serum levels of testosterone. The trial was terminated early as a result of an increase in adverse cardiovascular events among participants in the treatment group (26). The trial included both a self-reported measure, the Late-Life Function and Disability Instrument (LLFDI), and several performance-based measures including handgrip strength, bilateral leg and chest press (a measure of strength and power), 12-step stair climb, the 40-meter walk test, and the SPPB. The LLFDI assesses participants’ ability to complete discrete actions or activity and their performance of socially-defined tasks (activity and participation in the ICF framework).

Both anchor-based and distribution-based methods were used to determine the MCID for physical function. To assess anchor-based responsiveness, participants were grouped according to self-reported global rating of improvement (better versus no change or worse). The distribution-based responsiveness analysis provided an estimate of effect size, minimal detectable change based on a 90% CI (MDC_90_), and the percentage of participants exceeding MDC_90_ by group.

These analyses demonstrated that loaded walk and stair climb were the most sensitive, with anchor and distribution-based measures being similar. The SPPB balance assessment was the least sensitive measure. Handgrip strength and LLFDI were not responsive to change while both the Foundation of the National Institutes of Health (FNIH) and European guidelines advocate using handgrip strength to identify participants for sarcopenia trials (27, 28). These results suggest that this measure may be less useful to measure responsiveness to an intervention.

## Regulatory considerations of clinically meaningful change

Regulators prefer hard clinical endpoints to surrogate endpoints (e.g. biomarkers) when making decisions about market authorization. For example, in osteoporosis trials, a statistically significant difference in fracture rates – a hard clinical endpoint – is considered meaningful (29), whereas a surrogate endpoint such as bone mineral density would not in and of itself be considered meaningful, although it may be used to bridge studies for extension of indications.

The European Medicines Agency (EMA) guideline on clinical investigation of medicinal products used pain and function as co-primary endpoints in the treatment of osteoarthritis (30). The expert consensus committee that developed the guidelines suggested the threshold for minimal perceptible clinical improvement in pain as a 10 mm improvement on a 100 mm visual analog pain scale for drugs intended to improve symptoms or at least a 5 mm mean difference between placebo and active groups (31). These criteria were applied in a trial of chondroitin sulfate compared to placebo and the non-steroidal anti-inflammatory drug (NSAID) celecoxib, which showed that both drugs produced a statistically significant and clinically relevant improvement, yet whether the magnitude of the effect is sufficient to justify granting market approval remained an unanswered question (32).

A PROM, the SarQoL, has been developed to assess quality-of-life in sarcopenia patients (33). While it has demonstrated the ability to detect statistically significant change, the MIC has not yet been determined; thus, the clinical significance is not clear.

Whether to use continuous or dichotomous variables may also be discussed with regulators. For example, the FRActure in postmenopausal woMen with ostEoporosis (FRAME) study of the bone-forming agent romosozumab assessed percent change in BMD from baseline, demonstrating that the treatment results in a rapid increase in BMD in comparison to bone loss in the placebo group and at the same time reduces fracture risk (34). When using percent change the clinical significance of the observed absolute change must also be considered.

In addition to data on clinically meaningful change used to support marketing authorization for a treatment, payers and policy makers may require additional real-world data and cost-effectiveness studies to support reimbursement (35). For example, validation of the FRAX risk assessment tool was achieved by the Screening for Osteoporosis in Older Women for the Prevention of Fracture (SCOOP) study in the United Kingdom, which showed that screening with FRAX resulted in a reduced risk of hip fracture, i.e., that the tool is medically relevant (36). Another real-world study conducted by the French Ministry of Health – the Pharmaco-Epidemiology of GonArthroSis and coxarthrosis (PEGASus) study -- assessed the ability of multiple symptomatic slow-acting drugs for osteoarthritis to reduce the consumption of NSAIDs, which are associated with substantial adverse reactions. Only glucosamine sulfate showed a significant reduction in consumption of NSAIDs.

The FDA has a somewhat different perspective on meaningful change in that they focus on within-patient anchor-based change. Distribution-based approaches may be used as supportive or supplementary information. Moreover, they require changes to be meaningful to the patient, using terms to which patients can relate. This has led them to incorporate patient preferences into their deliberations and selection of outcome measures.

The Aging in Motion (AIM) coalition has been working with FDA for several years on a project to qualify gait speed alone and the SPPB as acceptable and endorsed measures of function. However, the agency has stressed the need for both an objective measure such as SPPB and a self-report approach, which has increased the complexity of the qualification process.

PROMs present many potential challenges for sponsors. The correlation between PROMs and objective performance measures is modest, and the FDA suggests using them together as joint outcomes. Multiple primary outcomes may increase trial sample size requirements. PROMs are also subject to differences in beliefs and behaviors, making them more difficult to compare across diverse populations. One suggested approach would be to use a goal attainment scale in which the patient sets goals as well as metrics for success.

PROMs, including QOL measures, also must be very specific to the indication. For sarcopenia, this means that PROMs should relate to how low muscle mass affects how patients feel, function, and survive. Used in combination with performance measures, they could provide a powerful way of demonstrating efficacy. While there has been a reluctance of pharmaceutical companies to embed context-specific PROMs in Phase 2 and 3 studies, doing so would produce an enormous body of data that could help establish relevant anchors to estimate MCID and validate other measures.

## Moving Forward

One problem for research into how the ICF guidelines are interpreted is that structure and function are typically assessed with clinical measures applied in a controlled environment while assessment of activity and participation require capturing the patient perspective, which is heavily influenced by the environment, adaptation mechanisms, resilience, and coping. Moreover, meaningful change is context, perspective, and purpose dependent.

The Task Force identified several key areas for future research that should be considered when setting the criteria for a clinically meaningful change in a clinical trial:

- Published estimates of MCID derived from study participants who are only mildly functionally limited may have limited value for studies that enroll participants at high risk of physical disability. In substantially impaired participants, a small improvement in a performance test may translate into a large benefit in daily life and be perceived by the participant. Future work should address MCID in subsets of the population stratified by ability, with the instruments chosen being appropriate for that level of ability.
- The validation framework described above offers a paradigm for thinking carefully about the ideal definition of clinically meaningful change and then working backwards to identify how to measure and assess meaningful change.
- To measure clinically meaningful changes in real-world performance, it may be appropriate to incorporate into trials continuous digital technologies such as accelerometers as well as novel analytical techniques to determine MCID, CMD, and MCII. Signal processing of accelerometer data may also identify additional features predictive of adverse or beneficial outcomes.
